# Oxidative stress-related patterns determination for establishment of prognostic models, and characteristics of tumor microenvironment infiltration

**DOI:** 10.3389/fsurg.2022.1013794

**Published:** 2022-11-01

**Authors:** Zihao Bai, Yihua Bai, Changzhong Fang, Wenliang Chen

**Affiliations:** ^1^ Graduate Department, Shanxi Medical University, Taiyuan, China; ^2^ Department of General Surgery, The 2nd Affiliated Hospital of Shanxi Medical University, Taiyuan, China

**Keywords:** gastric cancer, oxidative stress, tumor microenvironment, microsatellite instability, prognosis

## Abstract

Oxidative stress-mediated excessive accumulation of ROS in the body destroys cell homeostasis and participates in various diseases. However, the relationship between oxidative stress-related genes (ORGs) and tumor microenvironment (TME) in gastric cancer remains poorly understood. For improving the treatment strategy of GC, it is necessary to explore the relationship among them. We describe the changes of ORGs in 732 gastric cancer samples from two data sets. The two different molecular subtypes revealed that the changes of ORGs were associated with clinical features, prognosis, and TME. Subsequently, the OE_score was related to RFS, as confirmed by the correlation between OE_score and TME, TMB, MSI, immunotherapy, stem cell analysis, chemotherapeutic drugs, etc. OE_score can be used as an independent predictive marker for the treatment and prognosis of gastric cancer. Further, a Norman diagram was established to improve clinical practicability. Our research showed a potential role of ORGs in clinical features, prognosis, and tumor microenvironment of gastric cancer. Our research findings broaden the understanding of gastric cancer ORGs as a potential target for individualized treatment of gastric cancer and a new direction to evaluate the prognosis.

## Introduction

Gastric cancer is the fourth leading cancer with the highest mortality rate globally (1) and a considerable burden on society. In 2020 alone, approx. 760,000 people died of stomach cancer. Surgical treatment, systemic radiotherapy, chemotherapy, immunotherapy, and other therapies have been found beneficial to the treatment of gastric cancer. Still, due to the gastric cancer heterogeneity, the diagnosis is often made at the middle and advanced stage; thus, the therapeutic effect is not particularly effective. Nevertheless, identifying molecular subtypes of gastric cancer based on gene and transcriptome provides a basis for individualized treatment. In addition, the discovery of biomarkers guides immunotherapy and specific drugs treatment of gastric cancer (2). Oxidative stress can be caused by various reasons, such as ultraviolet radiation, smoking, drinking, intake of non-steroidal anti-inflammatory drugs, etc. Induction of oxidative stress causes ROS accumulation in the body, destroys cell homeostasis, leads to tissue damage, accelerates aging, and then participates in the occurrence of many diseases (3). Oxidative stress is known to play an important role in the occurrence of gastric cancer (4). However, the relationship of oxidative stress with the prognosis of gastric cancer remains largely unclear.

Identifying PD-1/PD-L1 and HER-2 as biomarkers in large cohort studies has helped immensely design the corresponding treatment strategies for clinical application (5). However, such studies are often based on a single biomarker without entirely satisfactory and convincing outcomes (6). Furthermore, previous studies on oxidative stress and gastric cancer have mainly focused on the effect of a single gene or single pathway (7). Thus, there is a growing need to construct a new prognostic marker based on molecular subtypes for the individualized treatment and prognosis of patients with gastric cancer.

In the present study, we aim to establish a scoring model (OE_score), through which patients with GC can be divided into high and low-risk groups for guiding treatment and assessment of prognosis. First, we clustered 732 GC patients based on the genes related to the prognosis of oxidative stress. This clustering revealed the subtypes related to prognosis and immune infiltration of GC. Then, according to the differentially expressed genes (DEGs) identified by these two oxidative stress subtypes, the patients were further divided into two gene subtypes. The model related to oxidative stress was established by the Lasso-Cox method, and thus OE-value was determined. This score was related to many characteristics, such as tumor mutation load, immunotherapy, microsatellite instability, etc. Our findings revealed a potential relationship between oxidative stress, prognosis, immune microenvironment, and immunotherapy response in GC patients. We have identified the potential relationship between oxidative stress and gastric cancer in the current study. In addition, a significant correlation exists between the overall effect of multiple ORGs on GC and the infiltration characteristics of TME. Meanwhile, the OE_score will help guide the individualized treatment of gastric cancer patients besides providing important insights for predicting the response of gastric cancer patients to immunotherapy.

## Methodology

### Acquisition and pre-processing of gastric cancer data resources

The gastric cancer transcriptome data (FPKM value) was downloaded, and corresponding clinical data were obtained from the TCGA official website (https://portal.gdc.cancer.gov/). As the TPM data is considered the same as the transcript from the microarrays (8), after transforming the transcriptomic data into TPM values, the data was merged with the chip data and clinical information of 357 gastric cancer tissues from the “GSE84433” dataset from the Gene Expression Omnibus (GEO) database (https://www.ncbi.nlm.nih.gov/geo/). The background adjustment and quantile normalization of the data were performed as the final data set. The batch effect caused by the non-biotechnology deviation was corrected using the “ComBat” algorithm of the “SVA” package. Patients without complete clinical information were excluded from the data.

### Survival analysis of oxidative stress genes

A total of 608 genes related to oxidative stress were obtained from the Amigo database (http://amigo.geneontology.org/amigo). In addition, 48 genes related to prognosis were screened by the univariate Cox regression and Kaplan-Meier analysis with the “survival” package and “survminer” package. The Log-rank test determined the difference in survival analysis. The adjusted *P*-value by the “LIMMA” package was <.001, indicating the statistical significance of gene for prognosis.

### Consensus clustering and gene set variation analysis (GSVA)

The number and stability of the obtained clusters were determined by the consensus using the clustering algorithm of the “ConsensuClusterPlus” package. Each subgroup after clustering had a certain sample size, and the samples within the group had a certain correlation. In contrast, the correlation between groups decreased after clustering. We used the “GSVA” R package to display and analyze the results of GSVA in a heatmap. The “C2.cp.kegg.v7.4.symbols” data obtained from MSigDB database was used for GSVA. In addition, single-sample gene set enrichment analysis (ssGSEA) was used to determine the level of immune cell infiltration in GC TME and the differences between the subtypes. The grouping effect was determined by principal component analysis (PCA) using the “ggplot2” R package.

### Clinical value of molecular subtypes in GC

The chi-square analysis of age, sex, T, and N stage was performed to obtain clinical information between the two subtypes. In addition, the Kaplan-Meier curve generated by the “survival” and “survminer” R package was used to evaluate the differences in RFS between different subtypes.

### DEG identification and functional annotation

By using the empirical Bayesian method of the “LIMMA” package, we obtained the DEGs. The adjusted *P*-value <0.05 and the fold-change of 1.5 were the screening criteria. We used the “clusterprofiler” R package to analyze the functional enrichment of these DEGs by GO and KEGG to explore the DEGs potential function.

### Construction of the oxidative stress-related prognostic OE_score

By calculating the model score of each patient, the grouping of each sample was obtained, and the corresponding treatment strategy was adopted. First, univariate COX regression was used to screen the DEGs related to the prognosis; then, based on DEGs, the molecular subtypes of GC patients were obtained using the same clustering and acquisition criteria as ORG subtypes. At last, through the “caret” R package, the GC patients were randomly into training (*n* = 364) and test groups (*n* = 364). We used the former to construct oxidative stress-related OE_score. Next, we used DEGs related to prognosis for constructing OE_score and used the “glmnet” R package to reduce the risk of overfitting. Finally, LASSO and multivariate Cox analysis selected the candidate genes to establish OE_score related to prognosis.

The OE_score was calculated as follows:

OE_score=Σ(Expi∗coefi)

In the formula, Expi and Coefi represent the expression level of each gene and the corresponding risk coefficient, respectively.

Based on this risk score, the patients were divided into high and low-risk groups according to the median score, and the prognosis was analyzed. According to the median risk score obtained from the training set, the total and the test set were divided into two subgroups, and the Kaplan-Meier survival analysis was carried out. The Log-rank test determined the difference in survival analysis. OE_score was evaluated by generating the receiver operating characteristic (ROC) curve, survival status, and risk scores distribution.

### Independence analysis and applicability of OE_score

Univariate and multivariate COX regression analysis was used to study the independence of OE_score. In addition, a stratified analysis was conducted according to the clinical characteristics of GC patients to determine the predictability of OE_score in different clinical groups.

### Determination of characteristic value of immunotherapy in OE_score-related subtypes

To obtain the difference of immune infiltration of subtypes, the CIBERSORT algorithm was used to quantify the score and infiltration of tumor-infiltrating immune cells in GC TME. The relationship between these 22 immune cells and the genes involved in constructing OE_score were explored. The ESTIMATE algorithm was used to calculate each patient's immune, stromal, and total scores. Further, the risk score was correlated with these scores. In addition, the relationship between these two risk groups and microsatellite instability (MSI), cancer stem cell (CSC), and tumor mutation load (TMB) was assessed. Finally, a boxplot was constructed to show the difference between the two groups of patients to determine the immunotherapeutic value of OE_score.

### Somatic mutation and analysis of chemotherapeutic drugs

The “maftools” R packet was used to process the mutation annotation format (MAF) obtained from the TCGA database to determine the similarities and differences of somatic mutations in GC patients between risk score subgroups. To treat patients in the scoring subgroup more effectively, we used a boxplot to visually present the semi-inhibitory concentration (IC50) value of chemotherapeutic drugs. These drugs concentration used to treat GC were calculated through the “pRRophetic” package, where a lower IC50 value depicts a more favorable chemotherapy regimen.

### Building a predictive nomogram

Based on the results of independent prognostic analysis, a predictive nomogram was built using clinical features and risk scores through the “rms” package. In the predictive line chart, the participating score variables of each sample match a score, and the total score obtained by each score can directly predict the 1-, 3-, and 5-year survival rate of the current sample (9). The calibration map of the predicted line chart was used to compare the gap between the predicted 1-, 3-, and 5-year ideal value and the real value to intuitively evaluate the prediction effect of the forecast line chart.

### Statistical analyses

All statistical analyses were carried out using the R version 4.0.3. The statistical significance was set as *P* < 0.05.

## Results

### Prognostic genes of ORGs in STAD

The analysis process of this study is shown in Figure S1. To explore the role of ORGs in GC, we integrated the TCGA STAD dataset with the expressive data. In contrast, the survival information from the GEO dataset was used to create a new dataset containing 732 samples for further analysis. The details of 732 patients with GC are shown in Supplementary Table S1. Using univariate Cox regression and Kaplan-Meier analysis, 48 ORGs were associated with the prognosis of GC patients. The expression of these genes is shown in Supplementary Table S2, and *P* < 0.001 was selected as the screening threshold.

### Expression of ORGs in STAD

**Figure 1:**
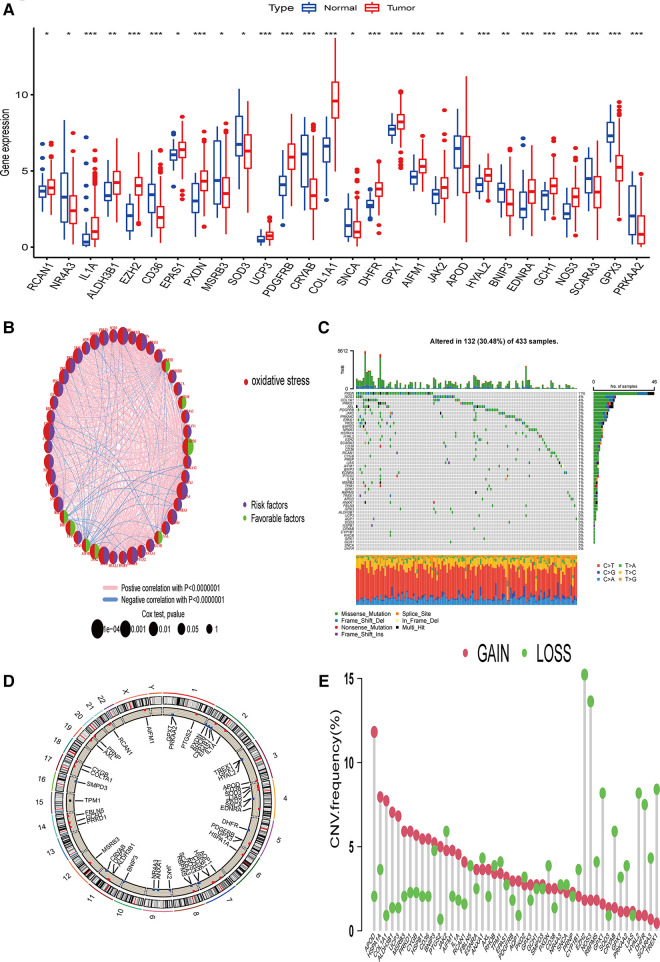
Genetic changes and ORGs gene expression in STAD. (**A**) Differentially expressed genes of ORGs in STAD issues and Normal tissues. (**B**) Interaction relationship of 48 ORGs in STAD. (**C**) Mutation type and mutation frequency of ORGs in STAD. (**D**) The location of ORGs in chromosomes. (**E**) The change of CNV of ORGs in the STAD cohort. ORGs, oxidative stress-related genes; STAD, stomach adenocarcinoma; CNV, copy number variant.

We first used the TCGA STAD dataset to study the differences in the expression of 48 prognosis-related ORGs between STAD and normal gastric tissues. In STAD, a total of 28 ORGs expressions were found to be up-regulated or down-regulated. More specifically, in the STAD group, the expression of RCAN1, IL1A, ALDH3B1, EZH2, EPAS1, PXDN, UCP3, PDGFRB, COL1A1, DHFR, GPX1, AIFM1, JAK2, HYAL2, EDNRA, GCH1, and NOS3 increased, while the expression of NR4A3, CD36, MSRB3, SOD3, CRYAB, SNCA, APOD, BNIP3, SCARA3, GPX3, and PRKAA2 were decreased (Figure 1A, *P* < 0.05). We also constructed a prognostic network map to directly identify the regulatory relationship between these ORGs (Figure 1B, *P* < 0.0000001).

### Genetic changes and ORGs expression in STAD

The incidence of copy number variation (SNV) and somatic mutation of 48 ORGs in STAD was summarized. As shown in Figure 1C, 132 (30.48%) of the 433 samples showed gene mutations. Among them, PXDN mutation frequency was the highest. In addition, we did not find any SNCA or DHFR mutations in any of the GC samples. T > A was the most common SNV type. Figure 1D shows the CNV changes on the chromosomes of the 48 ORGs. The frequency of CNV changes revealed that the 48 ORGs had general CNV changes. The amplification of CNV was mainly seen in APOD, while the loss of copy number mainly occurred in EZH2 and NOS3 (Figure 1E). Combined with the expression of mRNA of genes with obvious changes in CNV, the expression of APOD and MSRB3 was amplified by CNV decreased in GC, while the expression of ORGs amplified by CNV such as EZH2, NOS3, and DHFR was found to be increased in GC. The finding suggests that the change of CNV might be involved in the regulation of mRNA expression in ORGs. However, some genes amplified by CNV, such as HSPA1A, PRKD1, and other genes, did not differ in mRNA expression between the tumor and the normal group. This observation suggests that the change of CNV may be only one of the many factors that regulate the expression of mRNA of ORGs. While there are more factors such as RNA methylation, miRNA, lncRNA, and others that affect the expression of mRNA (10, 11). Our analysis showed significant differences in ORGs between the STAD and normal samples regarding genetic landscape or expression level. This data suggest that the overall effect of oxidative stress-related genes can affect the occurrence and development of GC. In addition, it might change the prognosis of patients by affecting somatic mutation and CNV.

### Identification of GC classification pattern mediated by 48 ORGs

**Figure 2:**
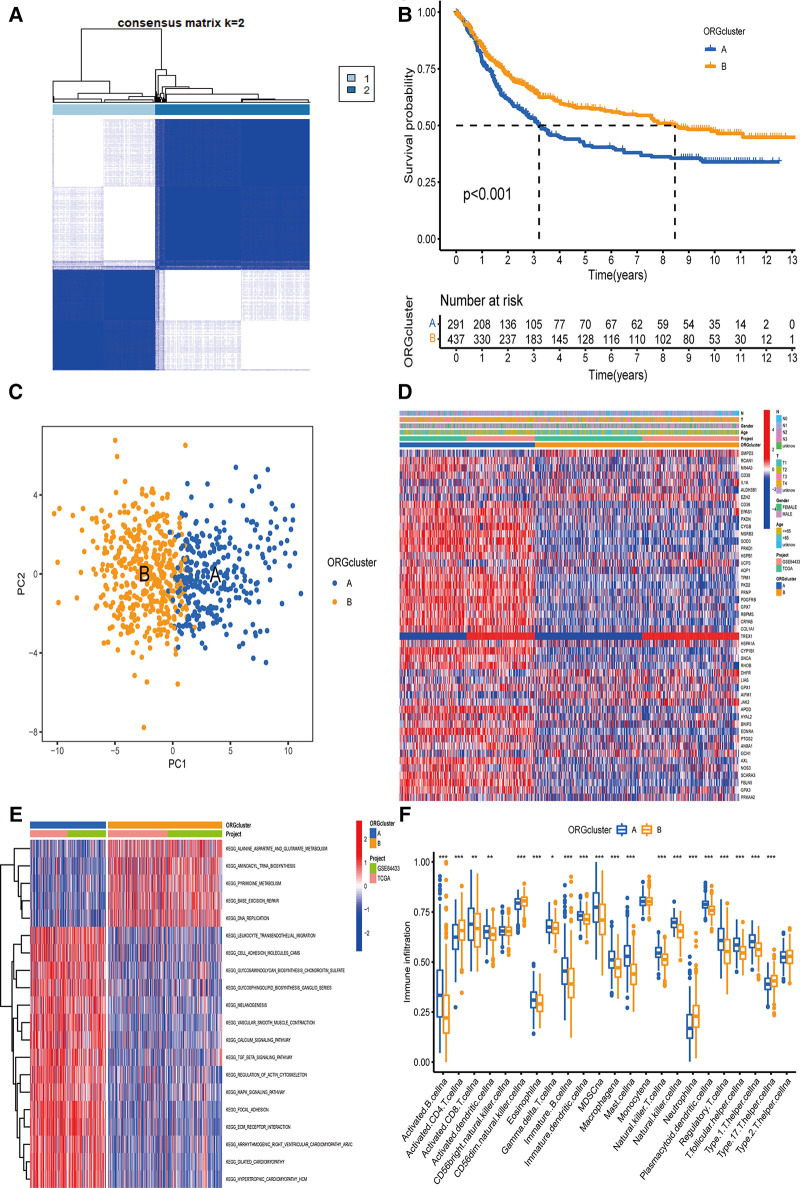
The determination of ORGcluster and the study of subtype function. (**A**) The patients with QC were divided into 292 cases in ORGcluster A and 440 cases in ORGcluster B by clustering algorithm. (**B**) PCA analysis of ORGcluster subtypes. (**C**) Differences in survival of ORGcluster subtypes. (**D**) Differences in clinical characteristics and gene expression of ORGcluster subtypes. (**E**) Study on the function of ORGs by GSVA. (**F**) Study on the difference of immune infiltrating cells of ORGcluster subtypes ORGcluster, the cluster of oxidative stress-related genes; GC, gastric cancer; PCA, principal components analysis; GSVA, gene set variation analysis.

Based on the expression levels of 48 ORGs related to prognosis, two ORGs were identified related to GC subtypes, including 292 cases in ORGs cluster group A and 440 cases in ORGs cluster group B (Figure 2A). The Kaplan-Meier curve revealed that the survival advantage of group B was significantly higher than group A (log-rank test, *P* < 0.001, Figure 2B). The PCA analysis revealed that these two subtypes could be distinguished significantly based on the expression of ORGs (Figure 2C). Heatmap arrangement showed that most of the genes were highly expressed in group A. Specifically, TPM1, PKD2, PRNP, PDGFRB, and GPX7 were significantly expressed in almost all the samples in group A, while SMPD3 and EZH2 were highly expressed in group B (Figure 2D). According to the GSVA, group B was significantly enriched in alanine, aspartate and glutamate metabolism, aminoacyl-tRNA biosynthesis, pyrimidine metabolism, DNA replication, base excision repair, and other pathways. In contrast, the group was significantly enriched in focal adhesion, ECM receptor interaction, dilated cardiomyopathy, hypertrophic cardiomyopathy (HCM), TGF beta signaling pathway, and calcium signaling pathway (Figure 2E). The enrichment of several extracellular matrix-related pathways suggests that oxidative stress may be related to the prognosis of patients by changing the content and composition of the matrix.

### Differences in TME infiltration characteristics between the two subtypes

The difference analysis of immune cells revealed that the expression of Type 17 T helper cell, Neutrophil, CD56dim natural killer cell, activated CD4 T cell was significantly higher in group B (Figure 2F). Furthermore, a significant difference was observed in the characteristics of TME cell infiltration between the two groups. T cells were associated with the infiltration of myeloid cells, making group B close to the immune-inflamed phenotype, while group A was more similar to the immune–excluded phenotype (12). In addition, these 48 ORGs can well distinguish the two subtypes.

### Acquisition of DEGs and determination of two gene clustering subtypes

**Figure 3:**
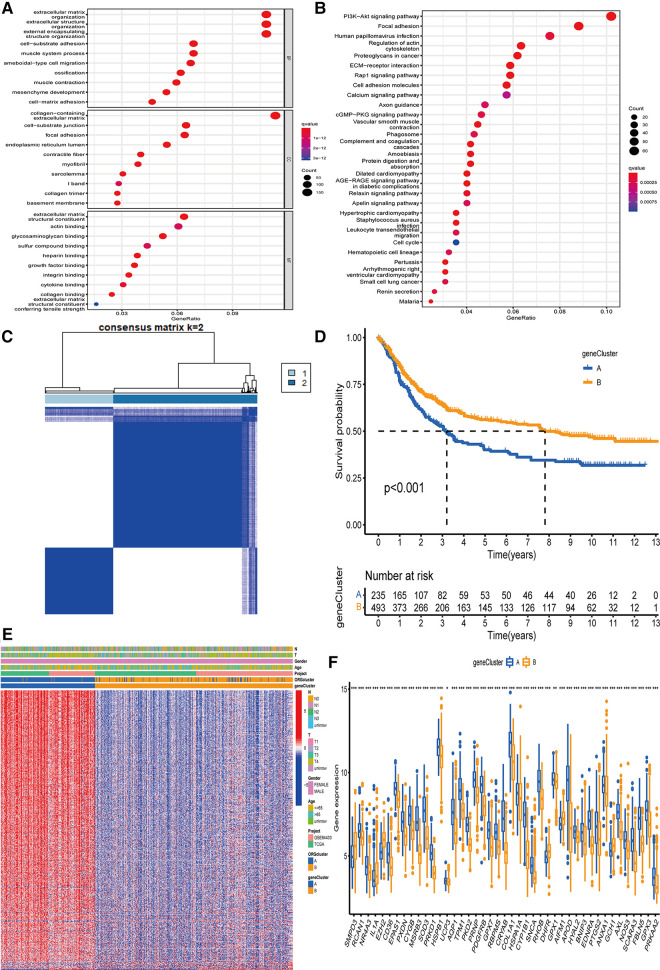
The determination of genecluster and the study of subtype function. (**A,B**) GO and KEGG on the enrichment of DEGs in difference pathways. (**C**) The clustering results of genecluster were divided into subtype A (*n* = 236) and subtype B (*n* = 496). (**D**) Survival analysis results of gene subtypes. (**E**) The difference in the expression of genes involved in the construction of the model between the two subtypes. (**F**) 43 genes involved in ORGcluster also showed differential expression in genecluster. GO, Gene Ontology; KEGG, Kyoto Encyclopedia of Genes and Genomes; DEGs, differentially expressed genes; ORGs, oxidative stress-related genes.

To better develop the clinical significance of two types of gastric cancer and develop an appropriate model for gastric cancer patients scoring, we explored the differential genes between the two subtypes and a specific genetic feature. We quantified the gene signature to apply it to the individualized treatment for GC patients. First, to identify the function of each oxidative stress mode, by analyzing the difference between the two subtypes, we obtained 1,358 DEGs related to oxidative stress subtypes. Then, we analyzed these genes using GO and KEGG databases. Our analysis revealed that related genes were significantly enriched in extracellular matrix-related biological processes, while in KEGG analysis, more genes were enriched in focal adhesion pathways (Figures 3A,B). Further, we screened out 593 genes that could be regarded as independent prognostic markers by univariate COX regression (adjusted *P*-value <0.05).

We used these 593 differential genes related to prognosis to construct the gene typing of patients with GC. The unsupervised clustering method identified two GC gene subtypes, including 236 cases in group A and 496 cases in group B (Figure 3C). The survival advantage of group B was significantly higher than group A (Figure 3D). The heatmap arrangement showed that almost all the genes involved in the grouping construction were highly expressed in group A (Figure 3E). Comparing the differential genes between the two groups showed that 43 of the genes involved in ORGs grouping were differentially expressed in gene grouping (Figure 3F).

### Construction of ORGs model

OE_score was established according to the oxidative stress-related DEGs. The data set was randomly divided into a training and a test set with 364 cases. We used the Lasso-Cox regression model to establish a characteristic score related to oxidative stress involving seven genes, named “OE_score”.

OE_score = (0.1378* expression of SLCO2A1) + (0.1025* expression of SHISA2) + (0.1034* expression of SERPINE1) + (−0.1752* expression of SMPD3) + (0.0727* expression of GPC3) + (0.0913* expression of CRABP2) + (−0.0856* expression of C1QTNF5).

**Figure 4:**
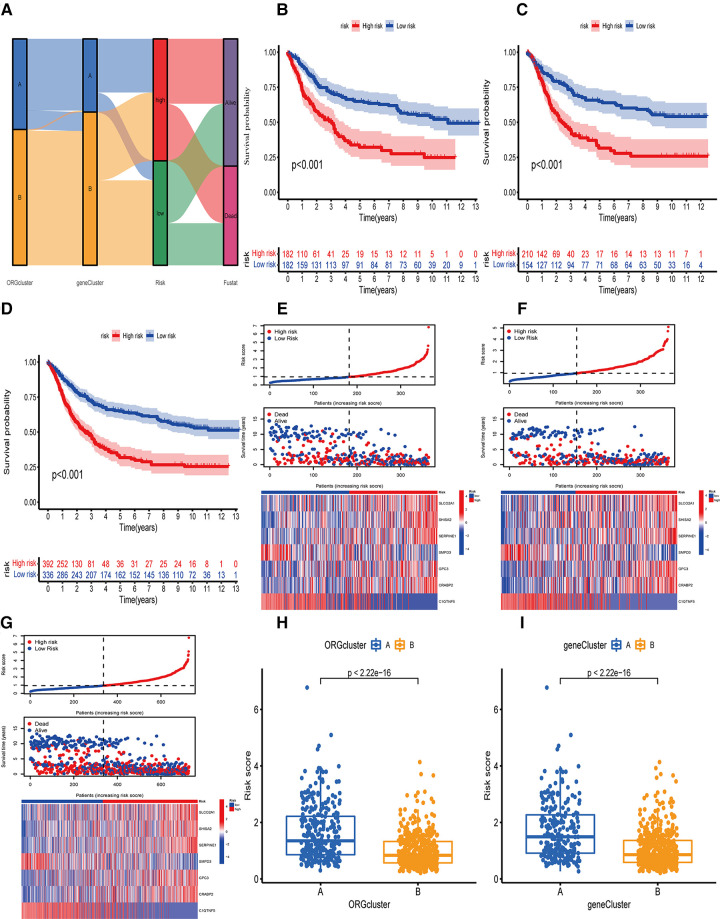
Survival analysis or OE_score in training set, test set and total data set. (**A**) Alluvial diagram of the distribution of the survival state of the samples of ORGcluster, genecluster and OE_score subgroups. (**B**) Survival differences between the two subgroups of the training group. (**C**) survival differences between the two subgroups of the test group. (**D**) Survival differences between the two subgroups of the total data set. (**E**) The risk score distribution and survival status in the training group, and the gene expression involved in the construction of OE_score. (**F**) The risk score distribution and survival status in the test, and the gene expression involved in the construction of OE_score. (**G**) The risk score distribution and survival status of the total data set, and participate in the construction of gene express of OE_score. (**H,I**) The difference of OE_score between ORGcluster and genecluster subgroups.

**Figure 5:**
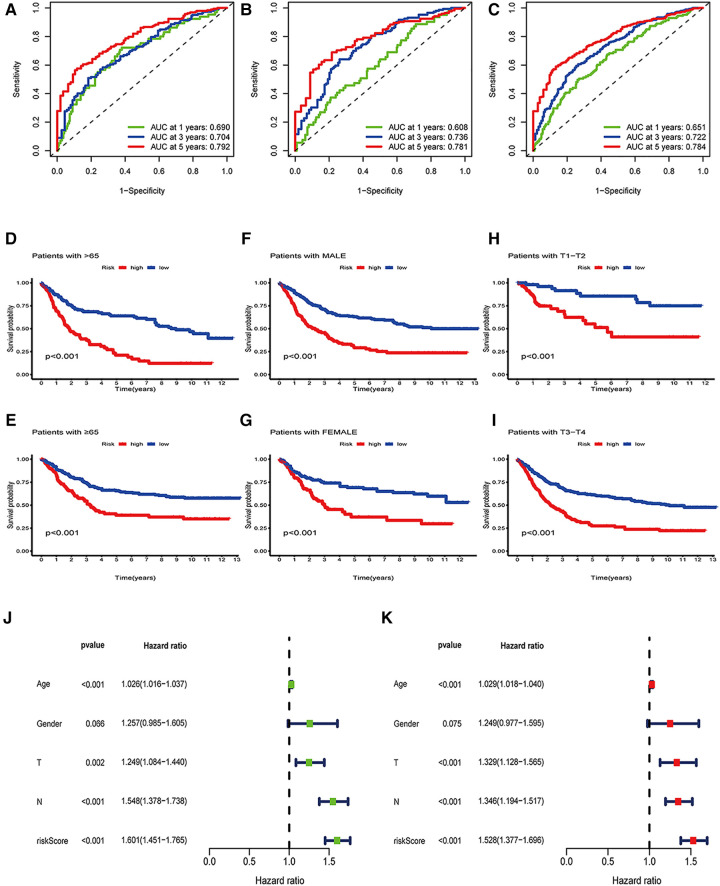
Independence analysis and hierarchical analysis of OE_score. (**A**) Using ROC curve to predict the sensitivity and specificity of 1-, 3-and 5-year survival rates based on OE_score in the training set. (**B**) Using the ROC curve, the sensitivity and specificity of predicting 1-, 3-and 5-year survival rates based on OE_score in the test set. (**C**) Using the ROC curve, the sensitivity and specificity of OE_score in predicting 1-, 3-and 5-year survival rates in the total data set. (**D–I**) Survival analysis of OE_score in high and low age groups, different gender groups and early and late T stage groups. ROC, receiver operating characteristic. (**J**) Univariate Cox regression analysis was used to determine that OE_score could be used as an independent factor affecting the prognosis of patients with gastric canser. (**K**) Multivariate Cox regression analysis was used to determine that OE_score could be used as an independent factor affecting the prognosis of patients with gastric cancer.

Further, we determined the value of OE_score by predicting the prognosis of patients. We divided the training patients into the high- and low-risk groups based on the median OE_score (0.949). The low-risk group had an obvious survival advantage (Figure 4B; *P* < 0.001). The low-risk group with the test and the total set had a better prognosis(Figures 4C,D; *P* < 0.001). The consistent distribution of risk scores with survival status indicated the general value of OE_score(Figures 4E–G). The test, training, and total set of these seven genes were expressed, as shown in Figures 4E–G. Meanwhile, the risk scores of ORGs typing and genotyping in group A were higher than group B (Figures 4H,I); this suggests that the subtypes with poor prognosis showed higher risk scores. There may be a correlation between OE_score and immune infiltration expression combined with prognostic analysis and immune infiltration. Therefore, next, we specifically analyzed the immune expression patterns and characteristics of OE_score. Further, our data revealed that OE_score was a good indicator for predicting 1 -, 3- and 5-year survival rates in patients with gastric cancer (Figures 5A–C). In addition, by incorporating the OE_score into the stratified analysis of clinical features, the score had good predictive ability in high and low age groups, different gender groups, and early and late T stage groups (Figures 5D–I). Thus, the OE_score could be used as a promising index to evaluate the prognosis of patients with gastric cancer. Figure 4A illustrates the survival state and distribution of the sample in two ORGcluster, two gene clusters, and high and low-risk groups.

### Building a predictive nomogram

**Figure 6:**
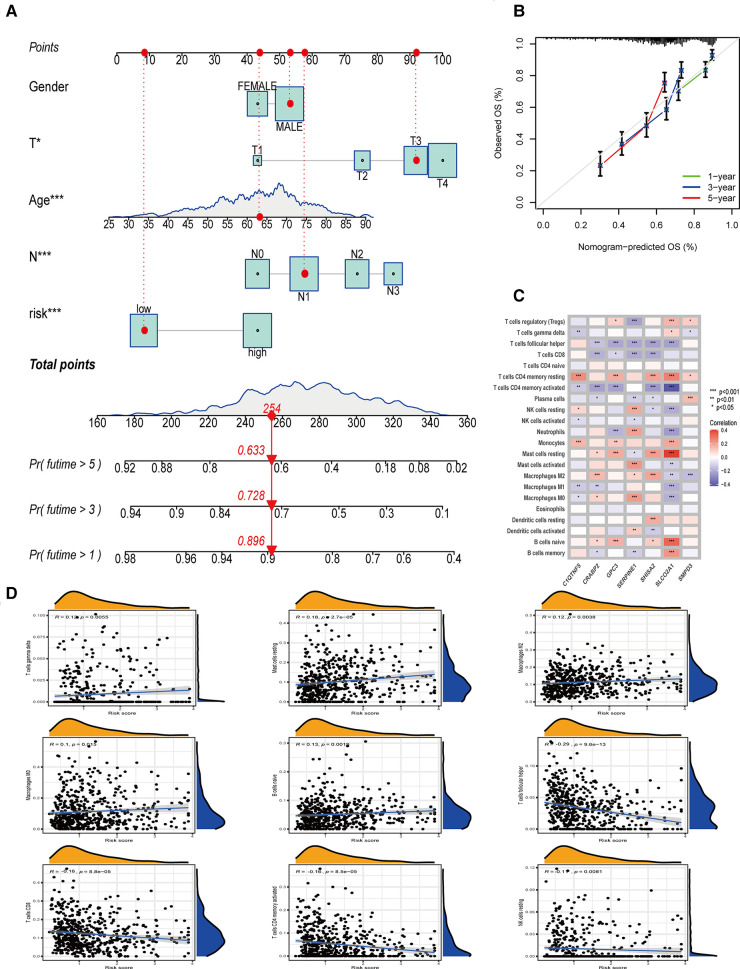
The establishment of nomogram, and the relationship between OE_score and tumor immune microenvironment. (**A**) Nomogram based on OE_score and other clinical factors to predict 1-, 3-and 5-year survival rates in patients with gastric cancer. (**B**) The calibration plot of the nomogram. (**C**) The relationship between the genes involved in the construction of OE_score and the expression of immune infiltrating cells. (**D**) The correlation between OE_score and immune infiltrating cells.

Combined with clinicopathological features and OE_score, a predictive nomogram is essential for clinical intuitive survival probability. The predictive nomogram was established by using independent factors affecting the prognosis of patients with gastric cancer, such as age, T stage, N stage, OE_score, and non-independent factors such as gender (Figures 5J,K, 6A). With the calibration chart, compared with the ideal model, the 3- and 5-year survival rates can be better predicted and applied in the clinic by combining the predictive nomogram of OE_score (Figure 6B). Furthermore, the good prediction of the survival of patients by predictive nomogram showed the rationality of constructing the OE_score, which is helpful to evaluate the prognosis of patients with GC.

### Relationship between ORG-related OE_score and immunotherapy in STAD

**Figure 7:**
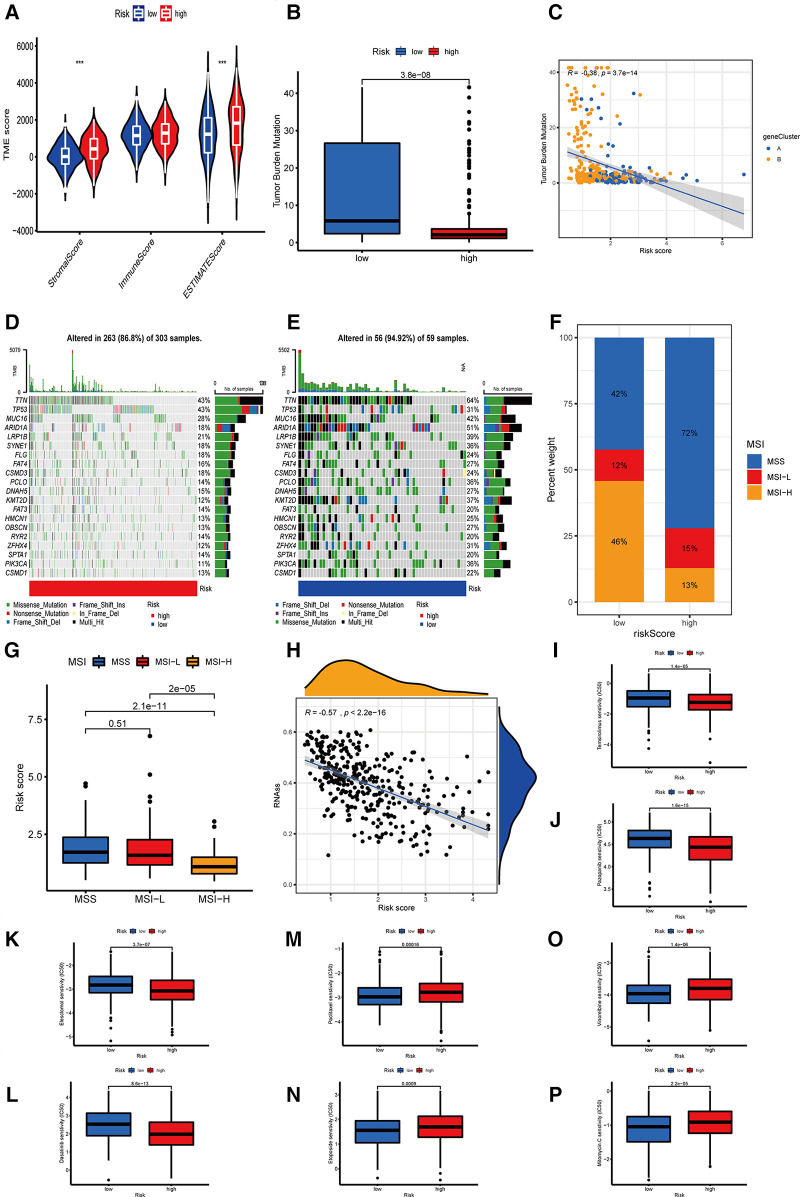
The relationship between OE_score and immunotherapy. (**A**) The correlation between the two OE_score-related subtypes and the TME score. (**B,C**) The correlation between OE_score and TMB. (**D,E**) OE_score high-risk and low-risk groups about the waterfall plot of TMB. (**F,G**) The correlation between OE_score and MSI. (**H**) the correlation between OE_score and CSC. (**I–P**) Sensitivity of patients with high and low risk of OE_score to various chemotherapautic drugs. TME, tumor microenvironment; TMB, tumor mutation burden; MSI, microsatelllite instability; CSC, cancer stem cell.

Oxidative stress plays a unique and important role in creating and maintaining the tumor immune microenvironment. Therefore, we decided to study the guiding value of OE_score for clinical treatment, especially immunotherapy. By analyzing the expression of genes involved in OE_score and immune infiltrating cells, we found that SERPINE1, SHISA2, and SLCO2A1 were strongly correlated with the expression of most immune infiltrating cells (Figure 6C). These genes might have caused the difference in immune characteristics of OE_score groups. In exploring and analyzing the relationship between OE_score and immune cells, a positive correlation was observed between OE_score and the abundance of T cells gamma delta (*R* = 0.12, *P* = 0.0055), Mast cells resting (*R* = 0.18, *P* = 2.7 × 10^−5^), Macrophages M2 (*R* = 0.12, *P* = 0.0038), Macrophages M0 (*R* = 0.1, *P* = 0.015) and B cells naive (*R* = 0.13, *P* = 0.0019). However, OE_score and the abundance of T cells follicular helper (*R* = −0.29,*P* = 9.6 × 10^−13^), T cells CD8 (*R* = −0.16, *P* = 8.8 × 10^−5^), T cells CD4 memory activated (*R* = −0.16, *P* = 8.5 × 10^−5^) and NK cells resting (*R* = −0.11, *P* = 0.0081) had the contrary result (Figure 6D). Tumors that attract more T cell infiltration are called “hot tumors” and are more sensitive to immunotherapy with better immunotherapeutic effects (13). The negative correlation between OE_score and multiple T cell infiltration suggests that the low-risk group might be close to our definition of “hot tumors” and was more suitable for immunotherapy to treat and delay the disease progression. Meanwhile, these data show a significant correlation between ORGs and tumor immune infiltration. The ESTIMATE algorithm (14) showed that the matrix score increased gradually with the increase of OE_score, while the tumor purity showed a contrasting effect. Still, no significant difference was found in the immune scores between the two groups (Figure 7A). The importance of stromal cells was reflected in all aspects of tumors, such as tumor growth, disease progression, and drug resistance (15–17). This suggests that our two subtypes had the GC heterogeneity through the difference of TME cells, thus affecting the outcome of treatment and prognosis.

Several studies have reported that TMB is a new biomarker for assessing the sensitivity of immune checkpoint inhibitors (18). In the present study, we found differences in TMB among different OE_score. The lower group had higher TMB, which indicated that the response to immunotherapy was better (Figure 7B). There was a negative correlation between OE_score and TMB (*P* = 3.7 × 10^−14^, Figure 7C). According to the waterfall chart, up to 94.92% of the 59 samples in the low-risk group had TMB, in which TTN and ARID1A had mutations in 50% of the samples, where missense mutations and multi-hit were the most common types of mutations. Among the 303 samples in the high-risk group, 86.8% had TMB, TTN, and TP53, with the highest probability of mutation (43%). The vast majority of mutation types were missense mutations. TP53 is an important gene involved in oxidative stress (19). The TP53 mutation rate (31%) in the low-risk group was lower than the high-risk group with a poor prognosis (Figures 7D,E). MSI is also considered a predictive biomarker of cancer immunotherapy (20). Patients with gastric cancer characterized by MSI-H tend to be more sensitive to immunotherapy, more suitable for related treatments, and exhibit a better prognosis (21). The patients in the low-risk group were characterized by MSI-H, while the patients in the high-risk group tended to show MSS (Figures 7F,G). One of the characteristics of MSI-H gastric tumors is the high level of CD8 ^+ ^T cell infiltration (22). This observation was consistent with our analysis, which might explain the effectiveness of immunosuppressive therapy at checkpoints in patients with MSI-H gastric cancer. Furthermore, we investigated the potential correlation of OE_score and CSC in gastric cancer. Figure 7H shows that OE_score was significantly negatively correlated with CSC index (*R* = −0.57, *P* < 2.2 × 10^−16^), suggesting that patients with low-risk scores had more obvious stem cell characteristics and low cell differentiation characteristics. Next, to explore the difference in the efficacy of chemotherapy drugs in the two groups of patients, the chemotherapy drugs currently used for the treatment of gastric cancer were used to explore the drug sensitivity related to OE_score. Interestingly, patients with high OE_score had lower IC50 values for Temsirolimus, Pazopanib, Elesclomol, and Dasatinib, while chemotherapeutic drugs Paclitaxel, Etoposide, Vinorelbine, and Mitomycin C had significantly lower IC50 values in patients with low OE_score (Figures 7I–P). Taken together, these results suggest that ORGs are associated with drug sensitivity. The analysis of OE_score based on ORGs, immune infiltration, and immunotherapy confirms that OE_score has a certain application value for assessing the effect of GC patients on immunotherapy. Moreover, it has potential significance for selecting treatment methods and assessing the prognosis results of GC patients.

## Discussion

Oxidative stress plays an important role in inflammation and tumor regulation (23); however, the TME and gastric cancer prognostic analysis remains unclear. The overall effect of multiple ORGs on GC and the characteristics of TME infiltration has not been elucidated. This study showed a correlation between the genetic landscape and the transcriptional level of ORGs in GC patients. Based on these 48 ORGs related to prognosis, we obtained two subtypes of ORGs with different clinical characteristics. The clinical features of type A were more obvious, with a worse prognosis. We obtained two gene subtypes based on the DEGs of two ORGs clusters. Our results showed that the ORGs might be an independent predictor of clinical outcome and immunotherapy response in GC. Based on this observation, an accurate and effective prognosis OE_score was constructed, proving its predictive ability. The oxidative stress patterns related to the occurrence and development of many diseases can be classified into high and low OE_score groups with different characteristics. Notably, there were significant differences in clinical characteristics, prognosis, mutation, TME, immune checkpoint, MSI, CSC index, and drug sensitivity between low-risk and high-risk patients with OE_score. Finally, we combined OE_score with tumor clinical characteristics to establish a quantitative nomogram, making OE_score widely used with a much easier approach. Through this score, we could directly predict the prognosis of patients, understand the occurrence and mechanism for the development of gastric cancer, and provide direct evidence for the treatment.

Although immunotherapy wide used to treat cancer patients has improved the survival rate in advanced stages (III/ IV stage patients). Still, a large number of patients show low responses to immunotherapy. These tumors generally lack lymphocyte infiltration in their microenvironment are often called “cold tumors” (24). Identifying these types of tumors and adopting corresponding treatment strategies might help decide the individualized treatment of tumors in these patients. A significant negative correlation was observed between OE_score and T cells follicular helper, T cells CD8, T cells CD4 memory activated, and NK cells resting. Of note, more CD8 ^+ ^T cells were expressed in the low-risk group. In a previous phase II trial of pembrolizumab, the CD8 ^+ ^T cells were associated with the resistance to PD-1 in MSI-H gastric cancer (25). With the increase of CD8 ^+ ^T cells, patients showed a better therapeutic effect. This observation was consistent with a better prognosis in the low-risk group of OE_score with high-MSI-H and high CD8 ^+ ^T cell infiltration. In routine clinical practice, there is a lack of peripheral markers analysis to reflect the efficacy of immunotherapy. The OE_score based on the variety of tumor immune infiltrating cells can determine which patients benefit more from immunotherapy. At the same time, a correlation was observed between peripheral immune cells and MSI, PD-1-related therapy.

The *γδ*T cells, a T cell subtype involved in the innate immune system, usually are double negative for CD4 and CD8 (26). This cell accounts for less than 5% of peripheral blood T cells and is associated with various inflammation and tumors (27, 28). In the study by Donnele Daley et al. (29), human pancreatic ductal adenocarcinoma (PDA) infiltrating *γδ*T cells were the main regulatory cells for *αβ*T cell activation. When *γδ*T cells are absent, many TH1 cells and CD8 ^+ ^T cells enter into TME and play an immune role. However, little is known about the interaction of *γδ*T cells with gastric tumors. However, a positive correlation was found between OE_score and *γδ*T cells. The differences in prognosis of high-risk and low-risk groups suggest that a large number of *γδ*T cells might be the reason for poor prognosis in high-risk groups. Moreover, *γδ*T cells can be used as a therapeutic direction for the outcome of the immunotherapy group. The *γδ*T cells have been used for the preparation of CAR-T and found therapeutically superior from the CAR-T prepared by *αβ*T cells (30).

Macrophages are one of the most important inflammatory cells in the tumor microenvironment. The Macrophages usually are of unpolarized M0 type and polarized classically activated macrophage (M1) and alternatively activated macrophage (M2) types. The infiltration of a large number of macrophages is often associated with the poor prognosis of gastric cancer (31). Therefore, we used macrophages as one of the prognostic markers. In ORGs classification, subtype A with a poor prognosis showed high expression of Macrophages. While a positive correlation was observed between OE_score and Macrophages M0, Macrophages M2. In-concurrence with previous findings, the high-risk group with poor prognosis expressed more Macrophages M0, Macrophages M2. Generally, M2 macrophages are polarized and participate in tissue repair and antiparasitic response (32). However, M2 macrophages exhibit an immunosuppressive effect in the tumor microenvironment, participate in matrix remodeling, and promote tumor growth and metastasis. Chen et al. (33) have found that CHI3L1 secreted by macrophage M2 can promote the metastasis of gastric and breast cancer cells both *in vitro* and *in vivo*. In addition, the expression of TGF-*β* affected the invasion of TAM and then the invasiveness of gastric cancer. PD-1 is also involved in the process of affecting the phagocytosis of macrophages and changing the tumor progression (34, 35). These studies have demonstrated the potential of monitoring Macrophages and their products as a diagnostic marker for gastric cancer. Of note, the use of depleted TAM or the conversion of TAM M2 to TAM M1 has been tried in anticancer therapy (36). This may also be an attempt to treat patients with higher OE_score.

Previous studies have shown that the mesenchymal stromal cells may participate in the polarization of M2 while promoting the metastasis and EMT of gastric cancer (37). Additionally, M2 macrophages are closely related to the extracellular matrix (ECM) and gastric cancer. The GO analysis of ORGs typing showed that the ORG-related subtypes were enriched in the ECM-related pathways. In KEGG, the focal adhesion pathway also had an obvious enrichment. In GSVA, the focal adhesion and ECM receptor interaction were also significant in expression pathways. The ECM is a complex collection of proteins, proteoglycans, and other molecules, while different tissues often have different structures and components. This difference gives functional and biological characteristics to the corresponding tissue. ECM is not simply involved in cell support and fixation; in gastric cancer, the role of the extracellular matrix has been proved to be involved in the process of disease initiation to metastasis. Importantly, the collagen gene in the focal adhesion pathway is a potential biomarker to distinguish gastric cancer from precancerous lesions (38). Oxidative stress induces ECM regulation and the interaction between oxidative stress. Thus, oxidative stress can be used as a potential target for treatment (39, 40). However, the effect of oxidative stress on ECM of gastric cancer is not clear. The interaction between extracellular matrix components and oxidative stress still has great potential as a biomarker and drug target for the prognosis of gastric cancer.

The risk score calculated by our scoring system was significantly related to the prognosis of GC patients and can be well distinguished in various characteristics. Based on the differences of TME, TMB, MSI, immunotherapy, stem cell analysis, and chemotherapeutic drugs, we can better distinguish the subtypes of gastric cancer patients. Moreover, this distinction can provide a new reference for individualized analysis and treatment of gastric cancer patients based on these gene expressions. In this study, seven genes (SLCO2A1, SHISA2, SERPINE1, SMPD3, GPC3, CRABP2, C1QTNF5) were used for the construction of OE_score, among which SLCO2A1, SERPINE1, CRABP2, and GPC3 were reported to be associated with gastric cancer (41–44), and play an important role in the occurrence and development of gastric cancer. The increased expression of SERPINE1 can promote tumor progression and angiogenesis by activating the VEGFR-2 signal pathway in gastric cancer (42). GPC3 has also been reported for the prognostic diagnosis of gastric cancer (45). These seven genes involved in constructing OE_score *in vivo* or *in vitro* can be explored further to study their potential regulatory relationship between upstream and downstream genes. The outcome might be useful for a new direction in treating and diagnosing gastric cancer.

Through *in vivo* or *in vitro* experiments, the relationship between genes involved in the construction of OE_score and gastric cancer will be assessed in the next step. Studying the relationship between these genes and the immune microenvironment will also provide important insight. Through single-cell sequencing, specific effects of oxidative stress on individual cells are also the focus of our future research.

This study had several limitations. Firstly, our data were obtained from the public database, and all the samples were retrospective in nature. There was a lack of verification of *in vivo* and *in vitro* experiments and large-scale randomized controlled trials to confirm our findings. Meanwhile, the effects of adjuvant radiotherapy and chemotherapy, neoadjuvant radiotherapy and chemotherapy, and surgical methods could not be fully considered in different cohorts. These limitations might have affected our judgment for assessing the relationship between oxidative stress and the prognosis of GC patients. It is worth mentioning that a variety of key enzymes leading to oxidative stress are involved in the production of reactive oxygen free radicals and active nitrogen free radicals. Meanwhile, antioxidant enzymes such as superoxide dismutase, catalase, and glutathione peroxidase are involved in the defense mechanism against oxidative stress. The variation in the coding genes of these enzymes (single nucleotide polymorphism, SNPs) affects the individual susceptibility to diseases, creates deviation in the gene expression database, and reduces the credibility of the results. In this paper, the biological process of oxidative stress is not analyzed from the point of view of SNPs, diet, physical activity, and several comorbidities. Moreover, these factors can easily affect the expression of oxidative stress genes, which might overshadow the individual differences, leading to biasness and data analysis limitations.

## Conclusions

In the current study, the predictive model based on oxidative stress-related genes along with the characteristics of immune infiltration was explored. In addition, the gene expression, clinicopathological and prognostic characteristics of ORGs cluster, gene cluster, and OE_score established by ORGs were studied.

The comprehensive analysis of ORGs unraveled their extensive relationship with the immune microenvironment, clinical features, and prognosis. The correlation between OE_score with seven genes based on ORGs and prognosis of gastric cancer patients with TMB, MSI, CSC, ECM, chemotherapeutic drugs were studied. The patients with low-risk scores had survival advantages in many aspects. These findings emphasize the potential role of ORGs in targeted therapy and immunotherapy based on patient's individual gene expression characteristics. Furthermore, the combined effect of multiple ORGs on the immune characteristics of gastric cancer is of immense value. The relationship between oxidative stress and gastric cancer is of great significance, providing a reference and basis for guiding the individualized treatment of gastric cancer. Moreover, it might enrich the existing ways of assessing the prognosis and choice of treatment of patients with gastric cancer.

## Data Availability

The original contributions presented in the study are included in the article/**Supplementary Material**, further inquiries can be directed to the corresponding author/s.
